# Mathematical appraisal of SARS-CoV-2 Omicron epidemic outbreak in unprecedented Shanghai lockdown

**DOI:** 10.3389/fmed.2022.1021560

**Published:** 2022-11-08

**Authors:** Minghao Jiang, Hongxin Yin, Shiyan Zhang, Guoyu Meng, Geng Wu

**Affiliations:** ^1^Shanghai Institute of Hematology, State Key Laboratory of Medical Genomics, National Research Center for Translational Medicine, Rui-Jin Hospital, Shanghai Jiao Tong University School of Medicine and School of Life Sciences and Biotechnology, Shanghai Jiao Tong University, Shanghai, China; ^2^State Key Laboratory of Microbial Metabolism, School of Life Sciences and Biotechnology, The Joint International Research Laboratory of Metabolic and Developmental Sciences, Shanghai Jiao Tong University, Shanghai, China

**Keywords:** SARS-CoV-2, Omicron variant, Shanghai lockdown, time-delayed differentiation equation model, quarantine ratio, Zero-COVID

## Abstract

The SARS-CoV-2 Omicron outbreak is ongoing in Shanghai, home to 25 million population. Here, we presented a novel mathematical model to evaluate the Omicron spread and Zero-COVID strategy. Our model provided important parameters, the average quarantine ratio, the detection interval from being infected to being tested positive, and the spreading coefficient to understand the epidemic progression better. Moreover, we found that the key to a relatively accurate long-term forecast was to take the variation/relaxation of the parameters into consideration based on the flexible execution of the quarantine policy. This allowed us to propose the criteria for estimating the parameters and outcome for the ending stage that is likely to take place in late May. Altogether, this model helped to give a correct mathematical appraisal of the SARS-CoV-2 Omicron outbreak under the strict Zero-COVID policy in China.

## Introduction

The SARS-CoV-2 Omicron variant was reported as a new variant of COVID-19 in November 2021 (1) and has broken out in Shanghai recently. It was once suggested that this variant should be renamed as SARS-CoV-3 because of its unique immunological characteristics and dramatic immune escape mechanism.^1^ Contrarily, though the pathogenicity and transmissibility of the Omicron variant is highly different from other SARS-CoV-2 variants, the sequence similarities between SARS-CoV-2 and Omicron sub-lineages are above 99.6%, which means that the original nomenclature should be followed (2). The Omicron variant carries mutations to help the virus to resist or escape immunity provided by COVID-19 vaccines (1, 3, 4). To prevent the spread of infection, the local government of Shanghai municipal decided to enforce a practical lockdown on 28 March. However, during the 15 days after the lockdown, there were still over 20,000 Omicron cases confirmed per day, including both diagnosed and asymptomatic. The situation was quite severe when compared to other places across mainland China during the same period or that in Shanghai several months ago. Meanwhile, the epidemic in Shanghai is declining now after the turning point, but forecast of the timeline of the epidemic is still needed for appropriate policy decisions as well as boosting public confidence. Here, we propose a new mathematical model to evaluate the epidemic trend of Omicron spreading in Shanghai and make forecasts for future.

## Results

### The prediction of the turning point of the epidemic

The Omicron epidemic in Shanghai has begun since early March 2022. Until 12 March, the number of confirmed cases per day did not exceed 100 (Supplementary Figure 1). Due to the surge in COVID-19 positives since mid-March, the Shanghai municipal government made the practical lockdown decision on March 28 to control the spread.

**FIGURE 1:**
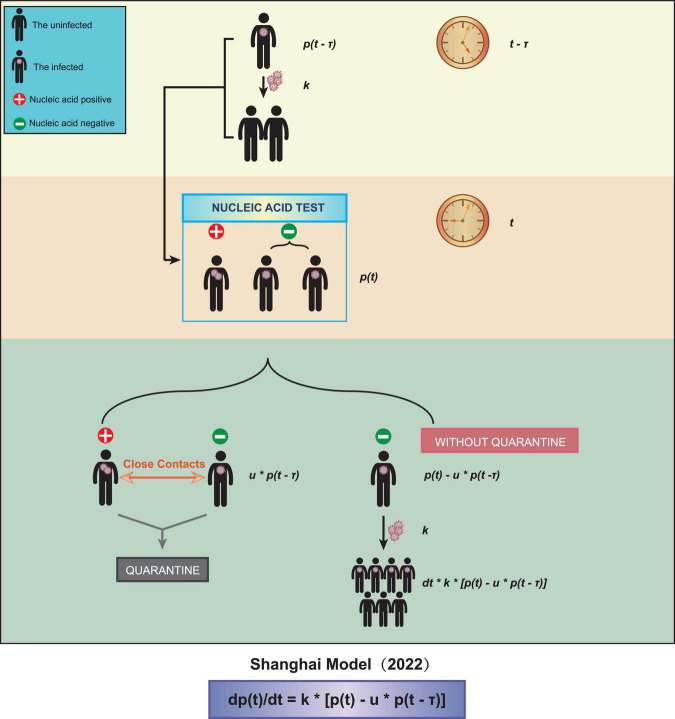
A schematic diagram of our model. Three parameters were taken into account, the test interval τ, the spreading coefficient *k*, and the quarantine ratio *u*.

We obtained a time-delayed differential equation to describe the spreading of COVID-19 Omicron (Figure 1):


$$\frac{d\,p\,\left(t\right)}{d\,t}=k\cdot \left[p\,\left(t\right)-u\cdot p\,\left(t-\tau \right)\right]$$

In the above equation, *p*(*t*) is the total number of patients infected at time *t*. The average spreading coefficient *k* represents the average number of people an unquarantined patient can infect in a unit interval. The average quarantine ratio *u* is defined as the number of quarantined patients divided by the number of people showing nucleic acid positive at time *t*. The average detection interval τ is the average time interval from being infected to being tested positive.

**FIGURE 2:**
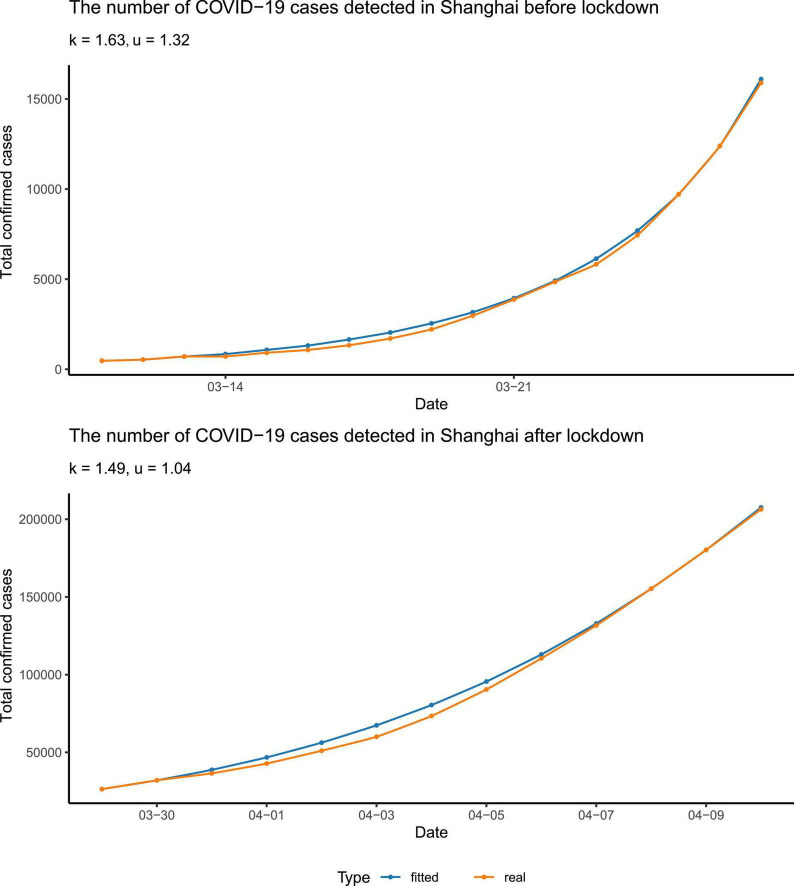
Data fitting. The data were fitted separately before and after the lockdown. The blue points and curve represented the reported number of total confirmed cases, and the yellow ones represented the number calculated from our differential equation. The parameters were rounded to three significant digits.

We fitted the data of the total number of reported cases before and after 28 March to our above equation, to figure out to what extent the more stringent policy could affect. Note that the date we used, 28 March 2022, was the very first beginning of the massive lockdown in the Pudong area, Shanghai, followed by the lockdown of the rest of the areas/districts in Shanghai on 1 April (Supplementary Table 5). Before 28 March, only a few communities had been officially controlled. We found that the fitting between the reported data and the curve calculated from our equation was quite close (Figure 2). We found that, to fit the data better, it was needed to alter the parameter τ in the two scenarios: (i) τ = 2 (days) before the city lockdown and (ii) τ = 1 (day) after the city lockdown. This can be construed as the more stringent quarantine policy after the city lockdown, with nucleic acid tests for all citizens once or twice per day, which reduced the detection interval τ.

In addition, we found that the spreading coefficient *k* decreased after the city lockdown, from 1.63 to 1.49, approximately (Figure 2). Furthermore, in the early stage of the epidemic, the quarantine ratio *u* was 1.32 > 1, which means that almost all the patients tested positive as well as their close contacts were quarantined. In the later stage of the epidemic, the quarantine ratio *u* became smaller, ∼1.04. The reduced *u* may serve as a sign of the arrival of the turning point. Since *p*(*t*), the cumulative number of confirmed cases, is a non-decreasing function, and its derivative, $\frac{d\,p\,\left(t\right)}{d\,t}$, has to be greater than or equal to zero. Therefore, from our equation, *u* must be smaller than or equal to $\frac{p\,\left(t\right)}{p\,\left(t-\tau \right)}$. At the beginning of the epidemic, Omicron was spreading quickly; *p*(*t*) was much larger than *p*(*t*−τ), so *u* was relatively bigger. When the turning point was getting close, Omicron spreading slowed down, thus *p*(*t*) was not much different from *p*(*t*−τ), and $\frac{p\,\left(t\right)}{p\,\left(t-\tau \right)}$ was close to one, which made the quarantine ratio *u* smaller than that in the early stage of the epidemic.

**FIGURE 3:**
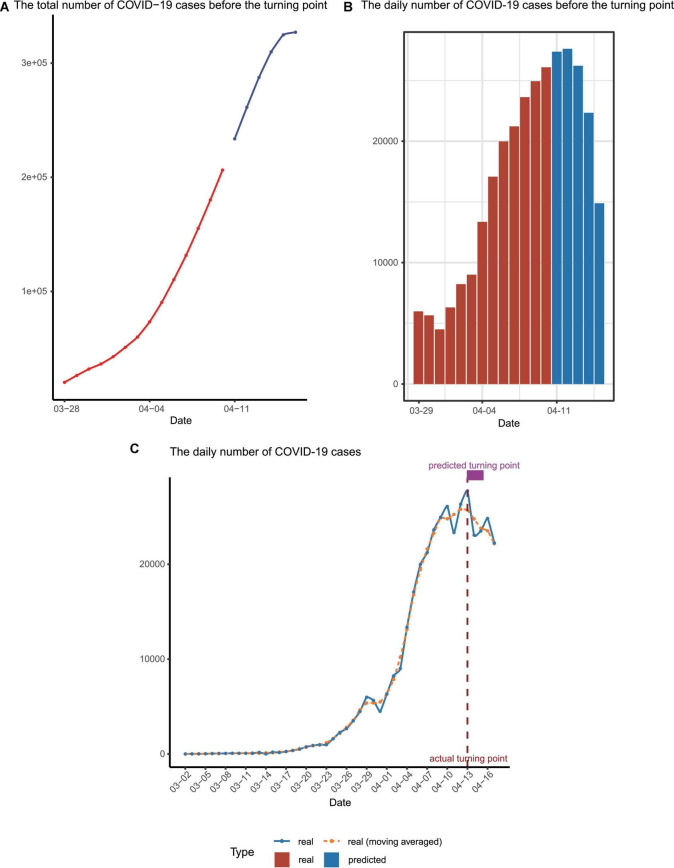
Epidemic trend forecast and validation. **(A)** The forecasted total number of confirmed cases before the turning point, with the predicted data points colored in blue. **(B)** The reported daily increased number of cases (red) and those for upcoming days predicted by our model (blue) before the turning point. **(C)** The number of infected cases per day. The dashed line represented the moving averages of the number of real cases while the solid line represented the number of real cases. The actual and predicted turning points were represented by the vertical dashed line and the rectangle above the curves.

With the three parameters estimated, the trend of Omicron spreading soon was predicted. We concluded that there will be more than 3,00,000 people infected in Shanghai during this Omicron outbreak (Figure 3A). Most importantly, we predicted that the number of confirmed cases per day would start to decrease around 13 April (Figure 3B), which was the so-called turning point.

We found that the average detection interval τ was 1 or 2 days when fitting the known data, which means that there would be 1–2 days of delay from infection to showing positive in the nucleic acid test, during which time, the unnoticed patients were still infectious (5–7). We believe that this is one of the major reasons why Omicron spread rapidly. Furthermore, the average quarantine ratio *u* was found to be greater than one, which means that most of the infected patients and their close contacts had been quarantined, indicating that the lockdown and other stringent policies were effective, making the appearance of the turning point and quelling the epidemic possible.

Moreover, the apparent R0 value, which is the number of people a patient could infect, could be obtained by multiplying the spreading coefficient *k* and the detection interval τ. After the lockdown, the value of R0 dropped from 3.27 to 1.49, providing further supporting evidence for the effectiveness of the lockdown.

We finished the forecast on 10 April and this manuscript/model was submitted to the online preprint server on 12 April (using infection data available up to 11 April) (8). Before our manuscript is submitted, the numbers of daily infected patients before 17 April were collected to check our prediction (Figure 3C and Supplementary Table 1). Strikingly, it turned out that the real turning point indeed appeared on 13 April and the date when the total infection number exceeded 3,00,000 is on 14 April, perfectly matching our mathematical prediction.

### The prediction of the final stage of the epidemic

**FIGURE 4:**
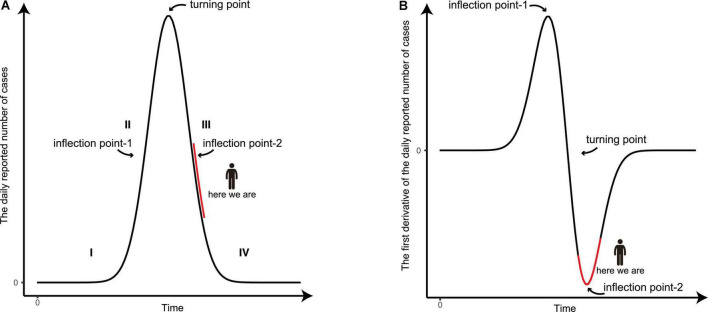
The graphic illustration of the development of an epidemic. **(A)** A schematic diagram of the number of the daily reported confirmed and asymptomatic cases. **(B)** The derivative of the daily reported number with respect to time during the development of an epidemic. The four stages of the epidemic are indicated and the present status of the epidemic in Shanghai is marked in red.

**FIGURE 5:**
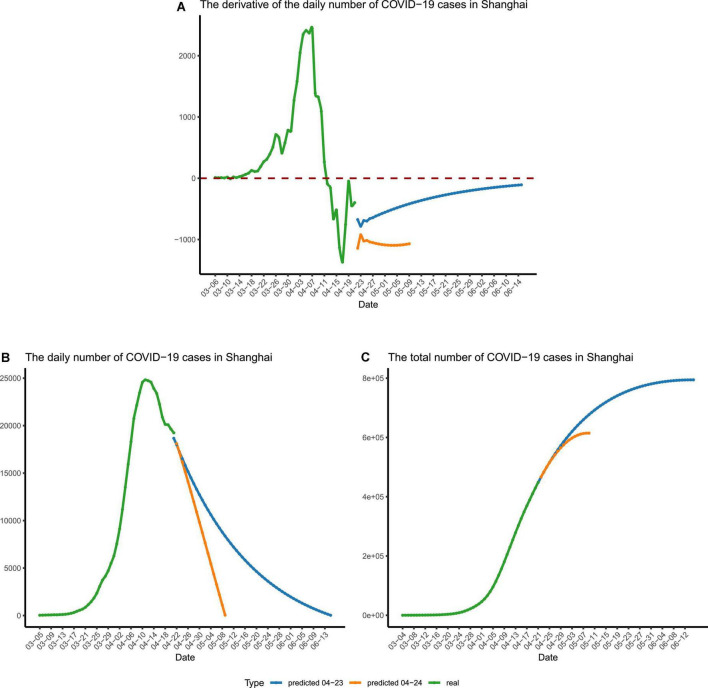
The forecast of the final stage of the epidemic. **(A)** The time derivative of the curve of daily reported cases in Shanghai. **(B)** The number of daily reported cases in Shanghai. **(C)** The number of recorded and predicted overall cases in Shanghai. The recorded numbers are colored in green. The predicted numbers are colored in blue and yellow, which were calculated using different sets of parameters. The blue lines represent the predicted numbers calculated using restrained parameters, and the yellow lines represent the predicted numbers calculated using relaxed parameters for stage IV.

It has been widely observed that an epidemic starting at a particular region cannot last forever, and the curve of the daily reported number of new infections generally has the shape of a bell-like curve (Figure 4; 5–7, 9), while the curve of total infections exhibits the shape of an S-like curve. At the beginning of the epidemic (stage I), the viral spreading accelerates rapidly and the slope of the infection curve increases significantly over time. As result, the second time derivative is positive and hence is recognized as a convex function. As the epidemic proceeds, it comes to a certain time point, coined as the inflection point-1 (Figure 4). In the period after the inflection point-1 (stage II), the slope of the curve of daily reported new infections starts to decrease with time. From this point onward, the second time derivative is negative and the curve turns into a concave function. When the turning point (*i.e.*, the slope of the curve is nil) is reached, the number of daily infections reaches its maximum level. This is a critical symbol/sign that the overall epidemic spreading is under control, as described in our previous report (8). From the turning point to the inflection point-2 (stage III), the number of daily infections declines rapidly, in which the second time derivative is still negative, still being a concave function. As the epidemic reaches its final stage after inflection point-2 (stage IV), the decline of daily infection starts to stabilize to a minimum level, in which the second time derivative becomes positive, changing back to a convex function. The two inflection points usually appear at half the maximum height of the curve. At the time we just finished the forecast, the COVID-19 epidemic in Shanghai is at the junction of phases III and IV because the derivative of the daily reported number in the last few days has been decreasing (Figures 4, 5A).

Meanwhile, taking into account the time variation of the three parameters in our mathematical model of the time-delayed differentiation equation is key to a relatively accurate long-term forecast. This can be understood as follows:

The total number of infected is a function of time, with three parameters *k*, *u*, and τ:


p=p⁢(t;k,u,τ)=k⋅[p⁢(t)-u⋅p⁢(t-τ)]

Therefore, we have


(1) $$\frac{d\,p}{d\,t}=\frac{\partial p}{\partial t}+\frac{\partial p}{\partial k}\,\frac{\partial k}{\partial t}+\frac{\partial p}{\partial u}\,\frac{\partial u}{\partial t}+\frac{\partial p}{\partial \tau }\,\frac{\partial \tau }{\partial t}$$

The equation (1) can be rewritten as:


$$\frac{d\,p}{d\,t}=\frac{\partial p}{\partial t}+\frac{\partial \,\vec{Param}}{\partial t}\cdot \nabla_{P\,a\,r\,a\,m}p=\left(\frac{\partial }{\partial t}+\frac{\partial \,\vec{Param}}{\partial t}\cdot \nabla_{P\,a\,r\,a\,m}\right)\,p$$

In the above equation, $\vec{Param}\;=\;(k,u,\text{τ})$ is a three-component vector and $\nabla_{P\,a\,r\,a\,m}=\left(\frac{\partial }{\partial k},\frac{\partial }{\partial u},\frac{\partial }{\partial t}\right)$ is the vector gradient. This equation is reminiscent of the well-known formula in fluid mechanics:


$$\frac{d}{d\,t}\,=\frac{\partial }{\partial t}+\frac{\partial }{\partial x}\,\frac{\partial x}{\partial t}+\frac{\partial }{\partial y}\,\frac{\partial y}{\partial t}+\frac{\partial }{\partial z}\,\frac{\partial z}{\partial t}\,=\frac{\partial }{\partial t}+\frac{\partial \,\vec{r}}{\partial t}\cdot \nabla =\frac{\partial }{\partial t}+\vec{v}\cdot \nabla$$

In other words, $\vec{Param}\;=\;(k,u,\text{τ})$ is equivalent to a moving frame of reference, similar to $\vec{r}\;=\;\left(x,\;y,\;z\right)$ in fluid mechanics (*e.g.*, a moving boat on a river). During a relatively short period, $\vec{Param}$ can be regarded as a constant vector (boat fixed in space), leading to $\nabla_{Param}=\vec{0}$ and $\frac{d\,p}{d\,t}=\frac{\partial p}{\partial t}$ (only the movement of the man on the boat needs to be considered). However, over a longer period, the frame of reference would change, so changes in $\vec{Param}\;=\;(k,u,\text{τ})$ along with time must be taken into account (both the movement of the man on the boat and that of the boat on the river need to be considered, Supplementary Figure 2).

To eliminate the jitter of the data, the moving averages (MA, *n* = 3) were calculated. For data fitting, the Python module gurobipy was used to carry out the calculation, and the sum of the absolute value of errors was minimized by solving quadratic programming. The three parameters, *k*, *u*, and τ, were obtained after fitting our differential equation to the MA number of daily reported cases from 11 April to 21 April, with *k* ≈ 0.66, *u* ≈ 1.00, and τ ≈ 1.5. Using the obtained parameters, the derivative of the number of daily reported cases is expected to reach the minimum on 4 May (Figure 5A). If the situation stayed the same afterward, the number of daily reported cases would drop below 1,000 by early 11 May (Figure 5B and Supplementary Table 2). However, we regard this as just an overly optimistic projection, and the reality may not be as good as expected, since the three parameters in our model vary with time and are not set in stone.

Therefore, to provide a relatively accurate prediction for the developing trend of the second half of the Shanghai COVID-19 epidemic, we have to take the time variation of the three parameters into consideration and switch to using the second set of parameters for the final stage (stage IV), during which a raised parameter τ and a shrinking *u* will be adopted.

Fitting our differential equation to the MA number of daily reported cases from 11 April to 20 April produced the parameters as follows: *k* ≈ 0.47, *u* ≈ 0.99, and τ ≈ 2. We chose this set of parameters to mimic an extended stage IV of the epidemic, with *u* getting smaller (*i.e.*, fewer people are quarantined) and τ getting bigger (*i.e.*, less frequent nucleic acid tests are carried out). Using these changed parameters, we predicted the ongoing epidemic trend of phase IV (Figure 5B). With the extended detection interval and reduced quarantine ratio, we forecasted that there would be more than 1,000 cases per day until early June and the zero-COVID status (*e.g.*, daily reported infections being less than 100) in Shanghai would be established by the middle of June (Figure 5B and Supplementary Table 2). Besides, this set of parameters forecasted that, during this epidemic, nearly 8,00,000 people would be infected (Figure 5C).

### The forecast of COVID-19 epidemic in Taiwan, China

**FIGURE 6:**
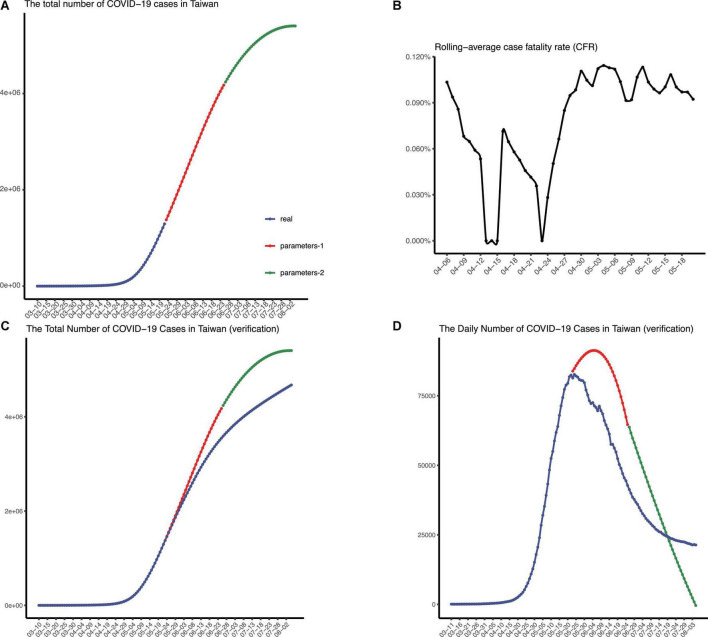
The epidemic situation in Taiwan, China. **(A)** The number of recorded and predicted overall cases in Taiwan, China. **(B)** The rolling-average case fatality rate in Taiwan, China. The validation of the prediction made in May, in terms of the number of total confirmed cases **(C)** and the number of daily confirmed cases **(D)**, respectively.

The population of Taiwan is similar to that of Shanghai, but the policy there was relatively loose. Since 7 March, the authorities approved the free entry of business people and reduced the duration of health monitoring. Around late May, the daily number of confirmed cases was over 80,000 in Taiwan. We forecasted the spreading of the pandemic with two sets of parameters (to mimic all four stages) in Taiwan, and it turned out, since 7 March, over 5,400,000 people would be infected in total (Figure 6A).

Next, the death toll in this pandemic was predicted. As the pandemic in Taiwan continued, the rolling-average case fatality ratio was calculated by the following formula:


R⁢o⁢l⁢l⁢i⁢n⁢g⁢a⁢v⁢e⁢r⁢a⁢g⁢e⁢C⁢F⁢R=7⁢d⁢a⁢y⁢s⁢a⁢v⁢e⁢r⁢a⁢g⁢e⁢n⁢u⁢m⁢b⁢e⁢r⁢o⁢f⁢d⁢e⁢a⁢t⁢h⁢s7⁢d⁢a⁢y⁢s⁢a⁢v⁢e⁢r⁢a⁢g⁢e⁢n⁢u⁢m⁢b⁢e⁢r⁢o⁢f⁢c⁢a⁢s⁢e⁢s⁢ 10⁢d⁢a⁢y⁢s⁢e⁢a⁢r⁢l⁢i⁢e⁢r

CFR equal to 0.9% was adopted finally, and we concluded that more than 4,800 people would die from this pandemic in Taiwan (Figure 6B). Deaths from COVID-19 were largely avoided by the lockdown in Shanghai, where only 587 COVID-19 deaths reported since 1 March.

As time goes on, we verified our prediction of the epidemic spreading in Taiwan (Figures 6C,D). We collected the data of daily confirmed cases in Taiwan after the date we first predicted and then plotted figures to check, which indicates that our model exhibited good performance in predicting COVID-19 spreading in Taiwan. Note that the epidemic wave beginning in March seems to reach equilibrium in early August (same daily confirmed cases every day). Additionally, 8,283 people died from COVID-19 in Taiwan from 7 March to 5 August.

## Discussion

The sudden Omicron epidemic outbreak in Shanghai has brought panic to the public, and a great loss to the business. To evaluate and forecast the spreading trend based on available confirmed data, we proposed a novel mathematical model, which took the local government policy (such as frequent nucleic acid tests and isolating those showing nucleic acid positive as well as their close contacts) into account, which could provide us important parameters describing the Omicron epidemic in Shanghai such as the spreading coefficient *k*, the quarantine ratio *u*, and the detection interval τ. The predicted number of overall cases and the expected time of turning point may help the government to make a judgment on the spreading and to revise the policies accordingly.

Several approaches that can be used to describe and predict the spreading of epidemics are deterministic, stochastic, and agent based (10, 11). Markov process can be used to model the possibilities of transition of individuals from one disease stage to another (compartments) in the stochastic framework (11). While, deterministically, ordinary differential equations (ODEs) can be applied to approximate the possibilities, which are sufficient to describe the spreading (12). There have been several fundamental/basic mathematical models that can be applied to epidemics (*i.e.*, mathematical epidemiology), for instance, the simplistic SIR model, the SIS model, the SEIR model, and the SEAIR model (11, 13). The simple theory relied upon by these models is that the population of an area could be divided into different compartments representing different stages of epidemic spreading. For example, three compartments are defined in the SIR model, *i.e.*, the susceptible (S), the infected (I), and the recovered (or dead) (R). Additionally, these models assume that individuals in the area have the same number of contacts and the same probability of contacting others, which is characterized by the homogeneous mean-field theory (14). In the SIR model, it is considered that the size of the population of an area (*N* = 1) is constant, which means: *N* = *S*(*t*) + *I*(*t*) + *R*(*t*), which represent the size of population of different compartment. Then, the following can be established:


$$\frac{d\,S\,\left(t\right)}{d\,t}=-\beta \,S\,\left(t\right)\,I\,\left(t\right)$$

$$\frac{d\,R\,\left(t\right)}{d\,t}=\gamma \,I\,\left(t\right)$$

$$\frac{d\,I\,\left(t\right)}{d\,t}=\beta \,S\,\left(t\right)\,I\,\left(t\right)-\gamma \,I\,\left(t\right)$$

β = μϕ, which is the product of the number of the infected population per person can meet per unit time and the infection probability of contact with an infected individual. Other basic ODE models take into account other compartments (SEIR) or assume different spreading processes (SIS). SIS premise is that there is no immunity forever for individuals and the infected would return to the susceptible stage again, while SEIR introduces the exposed stage into the whole system, where individuals ingest the pathogen but show no capacity to infect others (13). These models can provide essential information about the epidemic spreading, such as (1) R_0_ (basic reproduction number) without the estimation of the initial susceptible population; (2) The epidemic threshold, separating two phases of the epidemic; (3) The final epidemic size (FES); and (4) The endemic equilibrium (10–13). In addition, it can inform us how to reduce the contagion, for example, adequate pre-emptive vaccination coverage to the formation of herd immunity, reduction of μ, and minimization of ϕ. Importantly, these models could be fixed with complex networks to overcome the drawback that the demographic, economic, and other features are overlooked as epidemics affect cities and countries at the same time. Taking the Barabàsi-Albert (BA) model as an example, when new nodes are added to the network, their probabilities of connecting with others are proportional to degrees (15). The connectivity distribution denotes that hub nodes are difficult to spot, which is an important factor since hubs can easily cause massive spreading of the disease to neighbors, contributing to the spreading/infection speed. These models are still evolving and improving by different users. Global Prediction System of the COVID-19 Pandemic (GPCP) has been developed to forecast COVID-19 incidences on a seasonal basis in each country, based on the improved SIR (version 1) and SEIR (version 2) model (16). Zhang et al. proposed a novel stochastic model accounting for the transmission dynamics of COVID-19 in China (17). Unfortunately, the prediction of the epidemic spreading in Shanghai lacked precision.

Like these fundamental ODE models, the formula derivation and data fitting of our time-delayed differential model is based on the homogeneous mean-field theory and basic chain of epidemic spread, and some key parameters can be inferred from the model including the R_0_ value. In comparison, our model does not require the exact number of *S*(0) and *I*(0), but it models the spreading ability and quarantine ratio explicitly. Instead, the time interval τ is used to link the present confirmed cases and the reported confirmed cases τ day(s) before. Moreover, although the improved SIR and SEIR models can divide part of the population into the defined compartment, a range of equations and unknown parameters make its tough/tricky to handle and to predict the epidemic spreading easily. On the contrary, by emphasizing three essential parameters, especially for the description and prediction of the COVID-19 epidemic in China, our simpler model is much easier to use. Although our mathematical model is forged based on Shanghai’s anti-epidemic strategies which rely heavily on quarantine, it might be also applicable to other countries to some extent, whereas related parameters vary according to different government strategies and situations. Compared to western countries relying on the formation of herd immunity and mRNA vaccines which reduce the average spreading coefficient *k*, China attempts to boost the average quarantine ratio *u* to get the epidemic under control.

In addition, one prominent merit of our model, compared with other models such as those of machine learning, for example, is that our model can provide us with characteristic parameters of the epidemic, each of which has a distinct physical meaning (18). In contrast, models based on big data and machine learning are usually not physics-based and cannot reveal the rationale behind the prediction. For a better understanding, the parameters in our model represent different aspects in the real life. The average spreading coefficient *k* correlates with the mobility and concentration of the population and the vaccination percentage of the population, as well as people’s willingness to wear masks. A suddenly dropped *k* would mean tighter community control. In addition, the average detection interval τ was related to the frequency of nucleic acid testing. The more frequent the testing, the smaller the parameter τ would be. Finally, the average quarantine ratio *u* indicated that the proportion of people showing positive in nucleic acid tests and their close contacts who are quarantined. In the case of Shanghai COVID-19 epidemic (and presumably for other cities in China), stages II and III can be considered restrained states, with smaller values of *k* and τ. In contrast, stages I and IV can be considered as relaxed states, with looser parameters such as bigger values of *k* and *u*. To predict future trend of the epidemic, we would need to estimate parameters for stage IV. We propose that the criteria for making the estimation would be (1) the parameters for stage IV are more relaxed than those of stage III and (2) the parameters for stage IV are somewhat similar to those of stage I. Additionally, for further verification of our model, we also collected the data of confirmed cases in Jilin and Changchun cities, China, and predicted the turning point for each city by sets of parameters derived from fitting the data of the beginning of the epidemic, respectively (Supplementary Figure 4).

Our model, which considers the variation of the parameters, is easy to be applied to describe and forecast the spreading of the COVID-19 epidemic and other epidemic diseases and can help the society to avoid panic and build up the confidence to fight against COVID-19. We believe that our mathematical model presented in this report would help the public to have a better grasp of the current epidemic spread and would undoubtedly instill confidence and calm that are urgently needed in the caught-up fight against COVID-19 in China. However, at last, we want to make it clear to readers that the COVID-19 pandemic is unpredictable and our mathematical model for epidemic description is for Shanghai and related cities that are subjected to zero-COVID policy.

## Conclusion

We proposed, for the first time, a novel time-delayed differential equation to describe and forecast the spreading of the epidemic in Shanghai, by which we have predicted the turning point and the time when the number of infected would drop below 1,000 successfully. Altogether, our work was a novel mathematical appraisal of the SARS-CoV-2 Omicron outbreak and Zero-COVID policy in Shanghai lockdown.

## Materials and methods

### Data used in modeling

The case numbers were collected manually from officially released reports and moving averages were then calculated (Supplementary Table 3).

### Mathematical modeling

First, a differential equation was deducted to describe the spreading of COVID-19. Let us denote *s*(*t*) as the number of people whose nucleic acid tests are positive, then we have:


s⁢(t)=p⁢(t-τ)

In the above equation, *p*(*t*) is the total number of patients infected at time *t* and τ is the time interval from being infected to being tested positive. Since the patients infected during the time τ will not be recorded as nucleic acid positive, the diagnosed or recorded patients [*s*(*t*) above] at time *t* is equal to the number of infected population at time *t*−τ.

During the time *dt*, the number of newly infected patients *dp*(*t*) is:


d⁢p⁢(t)=k⋅f⁢(t)⋅d⁢t

The parameter *k* above is the spreading coefficient representing the average number of people an unquarantined patient can infect in a unit interval, and *f*(*t*) is the number of unquarantined patients at time *t*. The function *f*(*t*) is equal to *p*(*t*) subtracted by the number of quarantined patients *q*(*t*):


f⁢(t)=p⁢(t)-q⁢(t)

We further assume that *q*(*t*) (including both nucleic acid-positive patients and their close contacts) is proportional to *s*(*t*), and let *u* be the average quarantine ratio:


q⁢(t)=u⋅s⁢(t)=u⋅p⁢(t-τ)

Therefore, we get the final differential equation:


$$\frac{d\,p\,\left(t\right)}{d\,t}=k\cdot f\,\left(t\right)=k\cdot \left[p\,\left(t\right)-q\,\left(t\right)\right]=k\cdot \left[p\,\left(t\right)-u\cdot p\,\left(t-\tau \right)\right]$$

In this equation, there are three unknown parameters: the average spreading coefficient *k*, the average quarantine ratio *u*, and the average detection interval τ. These parameters may vary during different periods, for example, before or after the city lockdown. Using the number of reported cases during a particular time in Shanghai, these three parameters can be estimated by data fitting. As a next step, we could forecast the trend of Omicron spreading in Shanghai with these estimated parameters.

## Additional information

The forecast for the final stage of the epidemic, based on the number of cases from 11 April to 21 April, was finished and was submitted to online preprint server^2^ on 25 April, when the epidemic in Shanghai was at the stage III. Later, after getting to stage IV, we reanalyzed the up-to-date data to make clear the concordance of the forecast provided by our model. The restrained and the relaxed parameters were obtained by fitting the differential equation to the MA number of daily reported cases from 22 April to 29 April and from 22 April to 30 April, respectively. As shown in Supplementary Figure 3 and Supplementary Table 4, the number of daily reported cases would drop below 1,000 around 11 May and then drop below 100 around 17 May if the restrained parameters were adopted throughout, whereas the time when the infected population starts to fall below 1,000 and 100 were around 14 May and 30 May. Further, the just-released numbers of the infected on 14 and 15 May were 1,258 and 896, which still maintained the downward trend. In conclusion, the prediction of our model has a good consistency and was quite close to reality, and we believe the Shanghai epidemic will be under control soon.

## Data availability statement

The original contributions presented in this study are included in the article/Supplementary material, further inquiries can be directed to the corresponding authors.

## Ethics statement

Ethical review and approval was not required for the study on human participants in accordance with the local legislation and institutional requirements. Written informed consent from the patients/participants or patients/participants’ legal guardian/next of kin was not required to participate in this study in accordance with the national legislation and the institutional requirements.

## Author contributions

GM and GW conceived and designed the experiments. MJ performed the experiments. MJ, HY, SZ, GM, and GW prepared the figures and wrote the manuscript. GM supervised the project. All authors read and approved the final manuscript.

## Footnotes

1
https://www.science.org/content/article/new-versions-omicron-are-masters-immune-evasion

2
https://www.medrxiv.org

## Supplementary material

Supplementary Figure 1 The total number of confirmed cases in Shanghai from 1 March to the eve of the turning point. The blue points and curve represent the data before the official lockdown, and the red ones represent the data after the lockdown.

Supplementary Figure 2 A schematic diagram of the fluid mechanics’ formula and our mathematical model at the final stage of the epidemic prediction. All the three components in the fluid mechanics’ formula, the stream, the crew, and the ship were in flux, just as the three parameters in our mathematical model varied along the process of the epidemic.

Supplementary Figure 3 The forecast of the final stage of the epidemic when getting to stage IV. **(A)** The time derivative of the curve of daily reported cases in Shanghai. **(B)** The number of daily reported cases in Shanghai. The recorded numbers are colored in green. The predicted numbers are colored in blue and yellow, which were calculated using different sets of parameters. The yellow lines represent the predicted numbers calculated using restrained parameters, and the blue lines represent the predicted numbers calculated using relaxed parameters for stage IV.

Supplementary Figure 4 Data fitting and epidemic spreading prediction in Jilin and Changchun. Data fitting in Jilin **(A)** and Changchun **(B)** using the number of confirmed cases at the beginning of the epidemic in each city, respectively. The prediction of the turning point in Jilin **(C)** and Changchun **(D)**, respectively. In the data fitting, the yellow color represents the real data, and the blue color represents the number from the data fitting. In the prediction, the red color represents the real data and the blue color represents the prediction around the predicted turning point.
