# Supplementary material for: Mathematical appraisal of SARS-CoV-2 Omicron epidemic outbreak in unprecedented Shanghai lockdown
Source: Front Med (Lausanne). 2022 Nov 8;9:1021560. doi: 10.3389/fmed.2022.1021560 (PMC9679533; doi:10.3389/fmed.2022.1021560)
Supplement: Supplementary file 1 [file Data_Sheet_1.PDF]

Supplementary Table 1. The number of predicted and real infected cases around the turning point.

Predicted	Real total infected	Real daily reported	Real total infected (*)	Real daily reported (*)	Date
233705.4	229678	23342	230672	25253	04-11
261313.8	256008	26330	256469	25797	04-12
287521	283727	27719	282176	25707	04-13
309862.9	306799	23072	306944	24768	04-14
324750.5	330312	23513	330745	23801	04-15
327074	355132	24820	354272	23527	04-16
-	377380	22248	376442	22170	04-17

***These numbers were moving averages**
