# Supplementary material for: Mathematical appraisal of SARS-CoV-2 Omicron epidemic outbreak in unprecedented Shanghai lockdown
Source: Front Med (Lausanne). 2022 Nov 8;9:1021560. doi: 10.3389/fmed.2022.1021560 (PMC9679533; doi:10.3389/fmed.2022.1021560)
Supplement: Supplementary file 2 [file Data_Sheet_2.PDF]

Supplementary Table 2. The predicted timeline of the COVID-19 epidemic in Shanghai

Parameters	Daily reported number <10,000	Daily reported number <1,000	Daily reported number <100
current parameters	04-30	05-08	05-09
relaxed parameters	05-06	06-08	06-15
