# Supplementary material for: Mathematical appraisal of SARS-CoV-2 Omicron epidemic outbreak in unprecedented Shanghai lockdown
Source: Front Med (Lausanne). 2022 Nov 8;9:1021560. doi: 10.3389/fmed.2022.1021560 (PMC9679533; doi:10.3389/fmed.2022.1021560)
Supplement: Supplementary file 3 [file Data_Sheet_3.PDF]

Supplementary Table 3. The number of COVID-19 cases

Date	Daily infections (1, *)	Daily infections (1, **)	Daily infections (2, *)	Daily infections (2, **)
03-01	1	-	44	59
03-02	5	-	49	60.57
03-03	14	12.8	71	58.14
03-04	16	22.2	64	54.29
03-05	28	32.2	80	55.57
03-06	48	42.4	43	59.57
03-07	55	55.2	29	61.14
03-08	65	64.6	53	63.71
03-09	80	71.6	77	61.14
03-10	75	73.6	82	64
03-11	83	94	82	70.57
03-12	65	79.8	62	68.57
03-13	167	105.2	63	70.43
03-14	9	120	75	71.71
03-15	202	159	39	70.71
03-16	157	200.4	90	79.86
03-17	260	299.2	91	88.14
03-18	374	410.4	75	91.43
03-19	503	558.2	126	98.43
03-20	758	702.4	121	99.43
03-21	896	824.2	98	106.29
03-22	981	1045.4	88	115
03-23	983	1346.6	97	111.71
03-24	1609	1703	139	123.43
03-25	2264	2206.8	136	127.57
03-26	2678	2901.4	103	128.71
03-27	3500	3772.4	203	138.14
03-28	4456	4447	127	152.43
03-29	5964	4807.8	96	166.57
03-30	5637	5369.6	163	209.57
03-31	4482	6109	239	220.57
04-01	6309	6703.2	235	241.71

04-02	8153	8245.8	404	267.71
04-03	8935	10756.8	280	295.71
04-04	13350	13488.4	275	337.43
04-05	17037	16037.6	278	376.29
04-06	19967	18891.4	359	400.43
04-07	20899	21171.8	531	442.43
04-08	23204	22972.4	507	493.14
04-09	24752	23592.8	573	542.86
04-10	26040	24674.4	574	624
04-11	23069	25554.6	630	688.29
04-12	26307	25157.2	626	799.14
04-13	27605	24467.4	927	909.86
04-14	22765	24582.2	981	1013.86
04-15	22591	23599.8	1283	1134.71
04-16	23643	21967.2	1348	1292
04-17	21395	21087.8	1302	1513.29
04-18	19442	20176.8	1476	1809.86
04-19	18368	18945.4	1727	2177.57
04-20	18036	19116.4	2476	2584.71
04-21	17486	19331.4	3057	3137.29
04-22	22250	19379.6	3857	3671.86
04-23	20517	18974.8	4198	4330.29
04-24	18609	17939.4	5170	5250.29
04-25	16012	15442.2	5218	6457.57
04-26	12309	13332.8	6336	7664.71
04-27	9764	11450.2	8916	9226.43
04-28	9970	9685.6	11508	10926.71
04-29	9196	8584.6	12307	12731.71
04-30	7189	7734.6	15130	15131.43
05-01	6804	-	17072	17925.71
05-02	5514	-	17853	20565.14

1: Shanghai; 2: Taiwan, China

*Data source: Shanghai Municipal Health Commission (https://wsjkw.sh.gov.cn/swfb/index.html) and Global

**These numbers were moving averages
