# Supplementary material for: Mathematical appraisal of SARS-CoV-2 Omicron epidemic outbreak in unprecedented Shanghai lockdown
Source: Front Med (Lausanne). 2022 Nov 8;9:1021560. doi: 10.3389/fmed.2022.1021560 (PMC9679533; doi:10.3389/fmed.2022.1021560)
Supplement: Supplementary file 4 [file Data_Sheet_4.PDF]

Supplementary Table 4. The predicted timeline of the COVID-19 epidemic in Shanghai when getting to the stage IV

Parameters	Daily reported number <1,000	Daily reported number <100
current parameters	05-11	05-17
relaxed parameters	05-14	05-30
