# Supplementary material for: Mathematical appraisal of SARS-CoV-2 Omicron epidemic outbreak in unprecedented Shanghai lockdown
Source: Front Med (Lausanne). 2022 Nov 8;9:1021560. doi: 10.3389/fmed.2022.1021560 (PMC9679533; doi:10.3389/fmed.2022.1021560)
Supplement: Supplementary file 5 [file Data_Sheet_5.PDF]

Supplementary Table 5. The timeline of epidemic policies in March and April in Shanghai

Date	Policy
3-12	Online school teaching
3-15	Staggered commuting; Telecommuting
3-16—3-17	Massive nucleic acid screening of affected areas
3-18—3-20	Massive nucleic acid screening of other areas
3-26—3-28	Nucleic acid screening and/or short lockdown of all districts except Qingpu district
3-28	Lockdown of the whole Pudong area
4-1	Lockdown of the rest Puxi area
