# Supplementary figures and images for: Mathematical appraisal of SARS-CoV-2 Omicron epidemic outbreak in unprecedented Shanghai lockdown
Source: Front Med (Lausanne). 2022 Nov 8;9:1021560. doi: 10.3389/fmed.2022.1021560 (PMC9679533; doi:10.3389/fmed.2022.1021560)

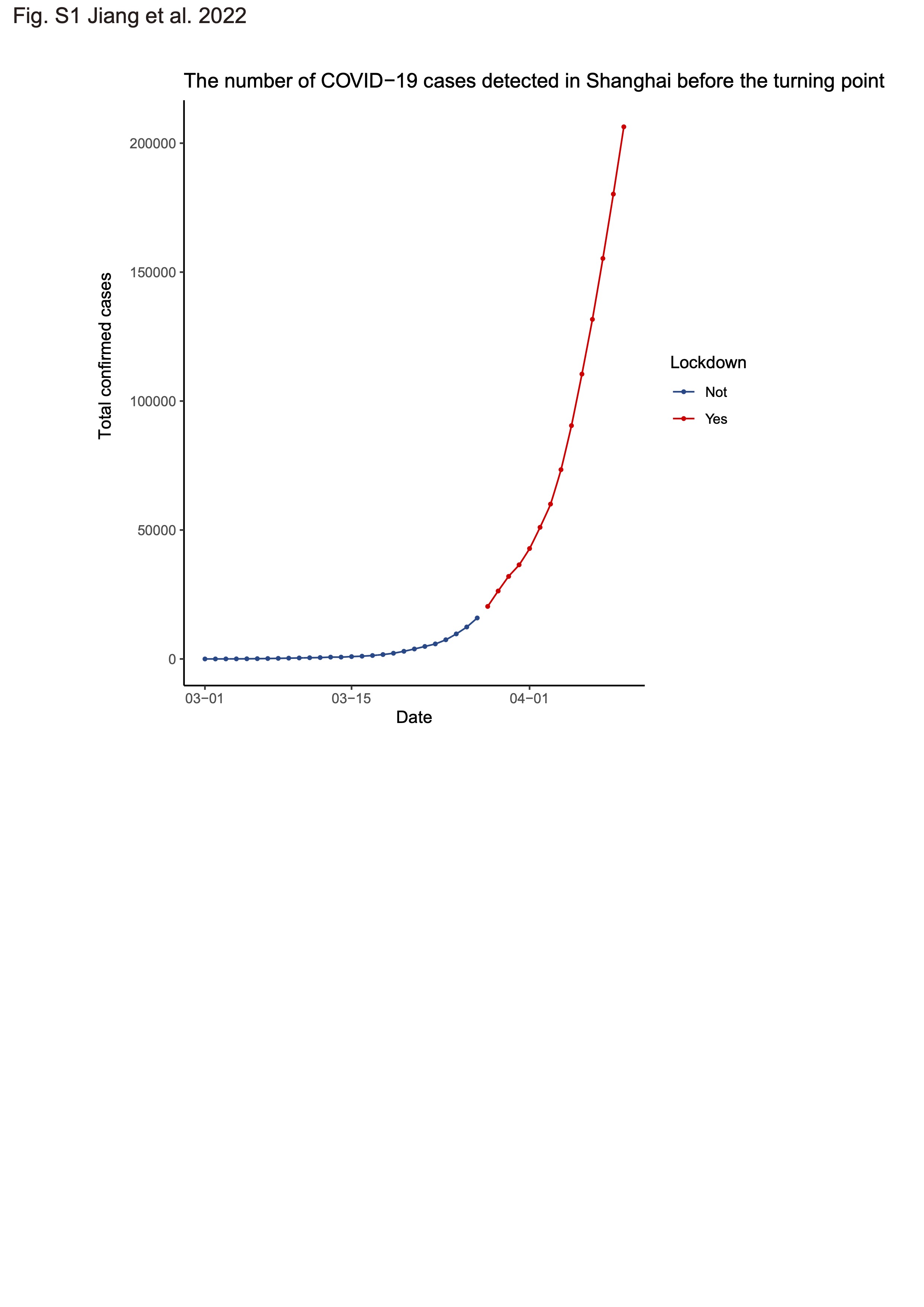

Supplement: Supplementary Figure 1 — The total number of confirmed cases in Shanghai from 1 March to the eve of the turning point. The blue points and curve represent the data before the official lockdown, and the red ones represent the data after the lockdown. [file Image_1.JPEG]

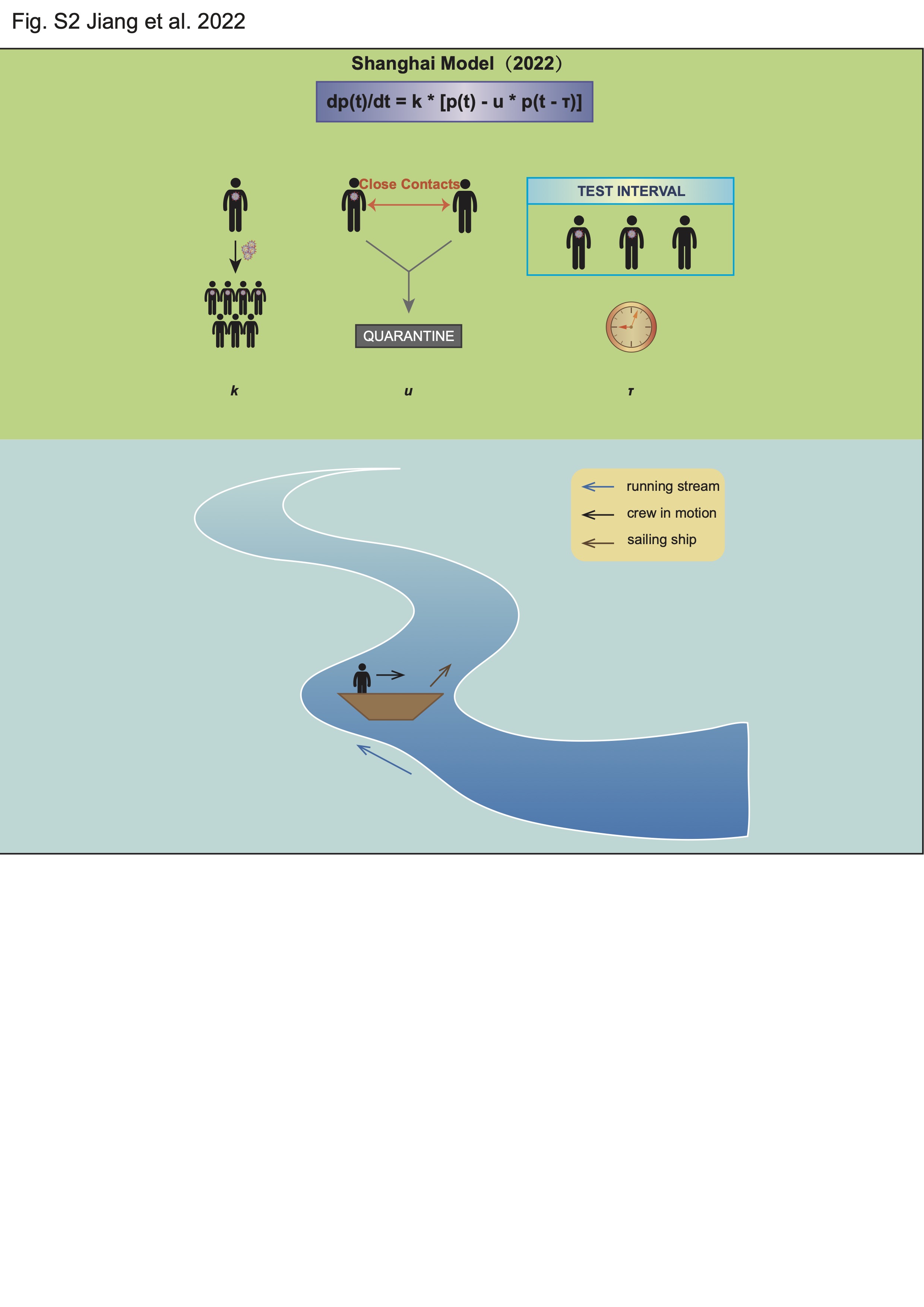

Supplement: Supplementary Figure 2 — A schematic diagram of the fluid mechanics’ formula and our mathematical model at the final stage of the epidemic prediction. All the three components in the fluid mechanics’ formula, the stream, the crew, and the ship were in flux, just as the three parameters in our mathematical model varied along the process of the epidemic. [file Image_2.JPEG]

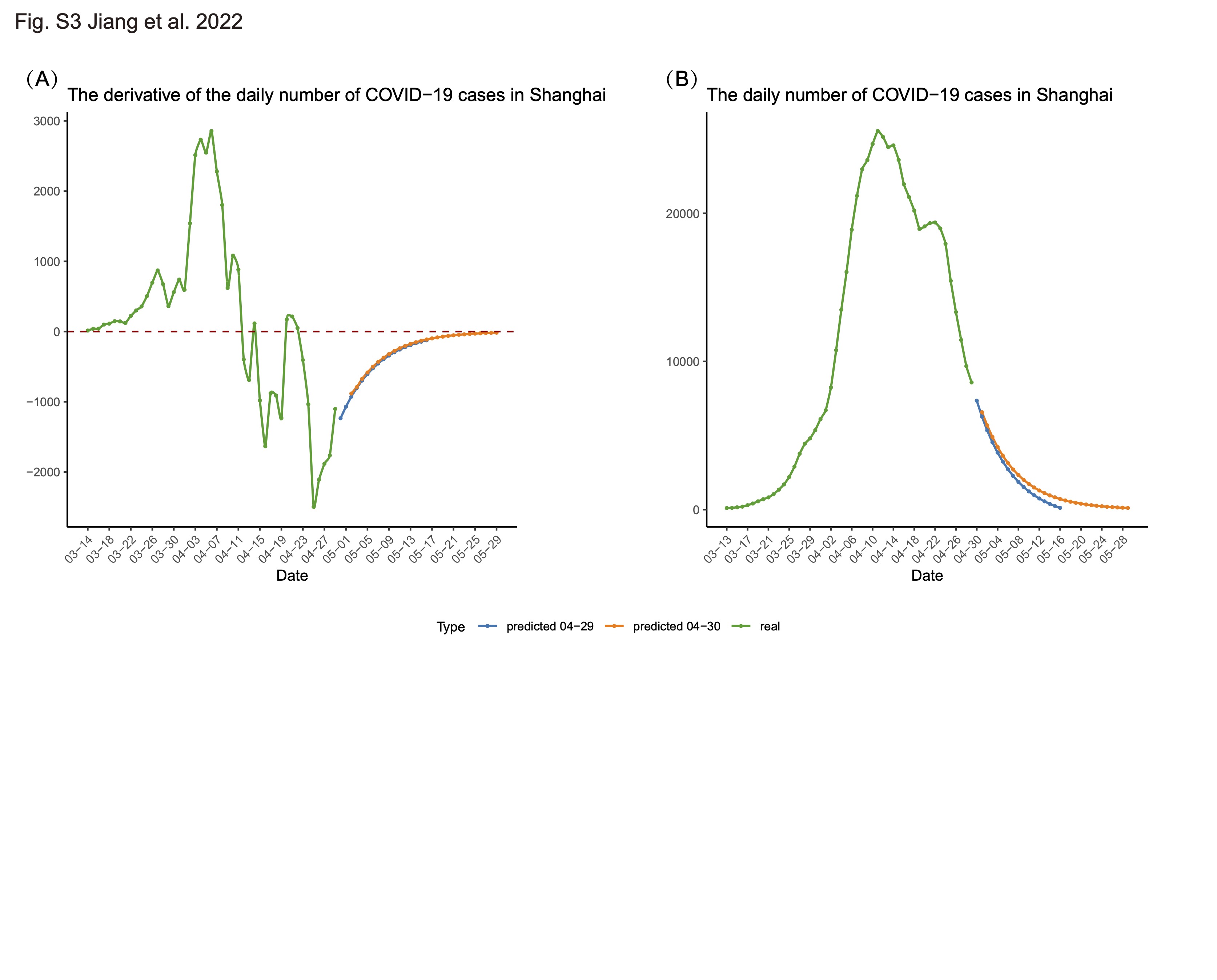

Supplement: Supplementary Figure 3 — The forecast of the final stage of the epidemic when getting to stage IV. (A) The time derivative of the curve of daily reported cases in Shanghai. (B) The number of daily reported cases in Shanghai. The recorded numbers are colored in green. The predicted numbers are colored in blue and yellow, which were calculated using different sets of parameters. The yellow lines represent the predicted numbers calculated using restrained parameters, and the blue lines represent the predicted numbers calculated using relaxed parameters for stage IV. [file Image_3.JPEG]

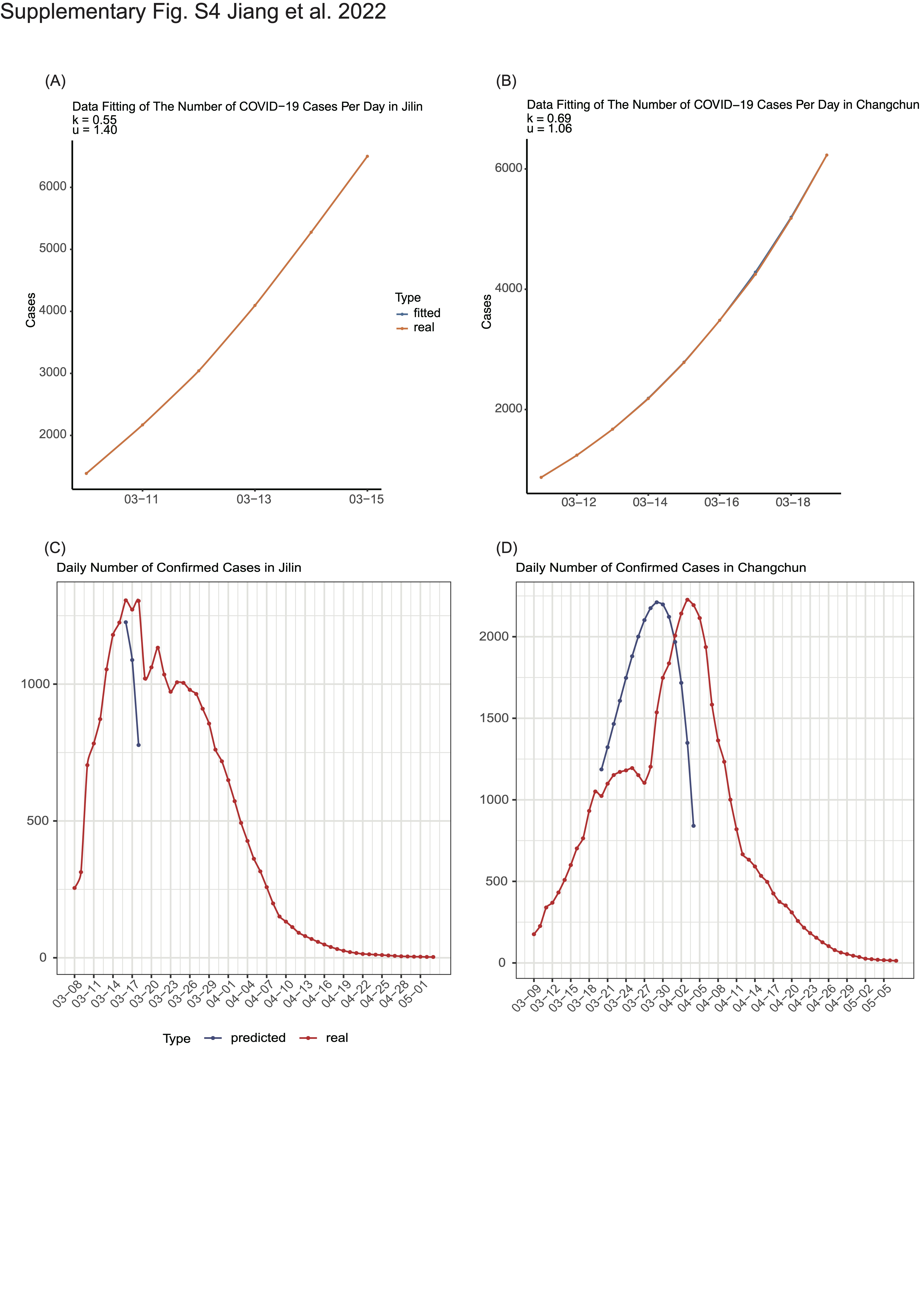

Supplement: Supplementary Figure 4 — Data fitting and epidemic spreading prediction in Jilin and Changchun. Data fitting in Jilin (A) and Changchun (B) using the number of confirmed cases at the beginning of the epidemic in each city, respectively. The prediction of the turning point in Jilin (C) and Changchun (D), respectively. In the data fitting, the yellow color represents the real data, and the blue color represents the number from the data fitting. In the prediction, the red color represents the real data and the blue color represents the prediction around the predicted turning point. [file Image_4.JPEG]
